# Psycho-social profile of patients with chronic neuropathic pain and spinal cord stimulation outcome: preliminary finding from an Italian sample recruited at the University Hospital of Verona (Italy)

**DOI:** 10.1192/j.eurpsy.2025.1897

**Published:** 2025-08-26

**Authors:** C. Perlini

**Affiliations:** 1 Department of Neurosciences, Biomedicine and Movement Sciences, University of Verona-AUOI of Verona, Verona, Italy

## Abstract

**Introduction:**

Spinal cord stimulation (SCS) is used to treat chronic neuropathic pain (CNP) resistant to other therapies and procedures. The treatment involves implanting a device that delivers electrical stimulation along the ascending nerve pathways. According to the bio-psycho-social model of pain, the success of SCS is influenced by more than just the technical aspects of the procedure. Psychological and context-related factors also play a crucial role.

**Objectives:**

To profile a sample of Italian patients with CNP in the pre-implant phase from a psycho-social perspective, using such data to predict the SCS outcome.

**Methods:**

Candidates for SCS at the Pain Therapy Center of the University Hospital of Verona (Italy) undergo a psychological assessment at the Clinical Psychology unit before the implantation. This assessment includes an interview to evaluate the impact of pain, coping strategies, and family support, as well as any history of traumatic experiences, psychiatric conditions, and lifetime use of alcohol or substances. Additionally, a series of questionnaires are administered to assess pain (Brief Pain Inventory, BPI), psychopathology (Symptom Checklist 90, SCL-90), personality (Minnesota Multiphasic Personality Inventory, MMPI-2), coping style (Coping Strategies Questionnaire, CSQ), the tendency to catastrophize (Pain Catastrophizing Scale, PCS), family and social support (Multidimensional Scale for Perceived Social Support, MSPSS), and self-efficacy (General Self-Efficacy Scale, G-SES). Patients are evaluated at 6 months follow-up (now ongoing).

**Results:**

To date, 131 patients (mean age 62.6±13.8; 56% females) have been evaluated at baseline. Overall, they show high percentages of somatization (71% of the sample), sleep disturbances (70%), depressive (40%), and obsessive-compulsive (38%) symptoms, together with moderate levels of catastrophizing (18.4±9.4 on the 0-36 range of the catastrophizing CSQ subscale), and a personality profile characterized by health worries (26%), somatic complaints (19%), and cynism (26%). Overall, they perceive a moderate level of self-efficacy (30.83±4.9 on the 0-40 range of the G-SES) and good family or social support (84%).

**Conclusions:**

Patients with CNP who are candidates for SCS show a peculiar psycho-social profile in terms of personality traits, coping strategies, and psychopathology. Using a pre-implant psycho-social assessment has significant implications for clinical practice since it allows to identify patients at a higher risk of SCS failure. It also enables the early detection of individuals who may benefit from psychological support before or after the SCS procedure.

**Disclosure of Interest:**

None Declared

